# A mixed-methods exploration into the resilience of community drug distributors conducting mass drug administration for preventive chemotherapy of lymphatic filariasis and onchocerciasis in Côte d’Ivoire and Uganda

**DOI:** 10.1371/journal.pgph.0000700

**Published:** 2022-07-15

**Authors:** Daniel Dilliott, David Addiss, Charles Thickstun, Adam Mama Djima, Esther Comoe, Lakwo Thompson, Stella Neema, Mary Amuyunzu-Nyamongo, Amos Wung-Buh, Deborah McFarland, Margaret Gyapong, Alison Krentel

**Affiliations:** 1 Bruyère Research Institute, Ottawa, Ontario, Canada; 2 NTD Support Center, Task Force for Global Health, Decatur, Georgia, United States of America; 3 School of Epidemiology and Public Health, University of Ottawa, Ottawa, Ontario, Canada; 4 Programme National de Lutte contre la Schistosomie, les Géo-Helminthiases et la Filariose Lymphatique, Ministère de la Santé et de l’Hygiène Publique, Abidjan, Côte d’Ivoire; 5 Onchocerciasis Control Programme, Ministry of Health, Kampala, Uganda; 6 Makerere University, Kampala, Uganda; 7 African Institute for Health and Development, Nairobi, Kenya; 8 Rollins School of Public Health, Emory University, Atlanta, Georgia, United States of America; 9 Center for Health Policy and Implementation Research, Institute for Health Research, University for Health and Allied Sciences, Ho, Volta Region, Ghana; The University of Texas Health Science Center at Houston School of Public Health - San Antonio Campus, UNITED STATES

## Abstract

Volunteer community drug distributors (CDDs) have been vital to progress made in the elimination of onchocerciasis and lymphatic filariasis; two neglected tropical diseases amenable to preventive chemotherapy (PC-NTDs). However, formative work in Côte d’Ivoire and Uganda revealed that CDDs can encounter considerable challenges during mass drug administration (MDA). CDDs must be resilient to overcome these challenges, yet little is known about their resilience. This mixed-methods study explored the resilience of CDDs in Côte d’Ivoire and Uganda. The characteristics and experiences of 248 CDDs involved in the 2018 MDAs in Côte d’Ivoire (N = 132) and Uganda (N = 116) were assessed using a micronarrative survey. Thematic analysis of CDDs’ micronarratives was used to identify challenges they encountered during MDA. Resilience was assessed using the Connor-Davidson Resilience Scale 25 (CD-RISC-25). Variables from the micronarrative survey found to be individually associated with mean CD-RISC-25 score (*P*<0.05) through bivariate analyses were included in a multiple linear regression model. Post-hoc, country-specific analyses were then conducted. Thematic analysis showed that CDDs encountered a wide range of challenges during MDA. The aggregate model revealed that CDDs who had positive relationships or received support from their communities scored higher on the CD-RISC-25 on average (*P*<0.001 for both), indicating higher resilience. These trends were also observed in the country-specific analyses. Mean CD-RISC-25 scores were unaffected by variations in district, age, gender, and length of involvement with the NTD program. Community support during MDA and positive community-CDD relationships appear to be associated with CDDs’ personal capacity to overcome adversity. Involving communities and community leadership in the selection and support of CDDs has the potential to benefit their well-being. This study establishes the CD-RISC-25 as a useful tool for assessing the resilience of CDDs. Further research is needed to understand, promote, and support the resilience of this valuable health workforce, upon which NTD programs depend.

## Introduction

Lymphatic filariasis and onchocerciasis are two parasitic infectious diseases that are classified by the World Health Organization as neglected tropical diseases (NTDs) [1]. Lymphatic filariasis (LF), resulting from infection with the filarial worms *Wuchereria bancrofti*, *Brugia malayi or Brugia timori*, causes lymphedema, elephantiasis, hydrocele in males, and the swelling of the genitals or breasts in females [2–4]. Onchocerciasis, a skin and eye disease caused by infection with the worm *Onchocerca volvulus*, is the second leading cause of infectious blindness globally after trachoma [2,3,5]. Both diseases are transmitted by insect vectors. Efforts to eliminate LF and onchocerciasis are based on mass drug administration (MDA), or the periodic distribution of preventive chemotherapy to eligible at-risk populations [6]. The World Health Organization’s 2030 NTD roadmap outlines these current strategies and milestones to reach elimination of both NTDs [7].

Volunteer community drug distributors (CDDs) are central to community-based MDAs. CDDs are responsible for preparing communities for MDA, increasing awareness and providing information about treatment, registering community members, and offering ‘mop-up’ treatment in low-coverage areas [8]. Through their efforts, billions of treatments have been delivered to people free-of-charge in NTD-endemic areas [9,10]. The African Program for Onchocerciasis Control (APOC) supported endemic countries to train and re-train millions of CDDs over the course of its efforts to control onchocerciasis [11]. Aside from distributing medications, the ability of CDDs to share information and encourage community members to participate in MDA is essential for the program’s success [12–14]. When CDDs are perceived as knowledgeable, professional and performing their job well, communities trust the program and are more willing to accept treatment [14,15]. However, when CDDs are perceived as lacking knowledge, are unprofessional or perform poorly, community members are less likely to participate [12]. As such, supporting CDDs with the training, skills and resources they need to be motivated and to perform their job well is crucial for achieving global elimination targets [14–17]. A well-supported, highly trained, and community-based volunteer workforce forms the basis of all three pillars of the WHO’s 2030 NTD roadmap (accelerating programmatic action, intensifying cross-cutting approaches, and changing operating models and culture to facilitate country ownership) [7].

During the formative phase of this project, CDDs in Côte d’Ivoire and Uganda revealed that they experience unexpectedly high levels of trauma, stress, divided loyalties and moral distress, similar themes to what has been reported by humanitarian aid workers [18–22]. CDDs may experience tension between their role as community members and as representatives of the global disease control initiatives. Traumatic and stressful experiences encountered by CDDs were evident in the formative research including stories about mistakenly treating pregnant women, being verbally abused by drunken community members, being frightened or chased from households by dogs or community members, and having doors slammed in their faces. The current paper explores CDD resilience as they encounter these stressors while carrying out their work.

Earliest conceptualizations of resilience focus on a strengths-based approach, and has been largely attributed to Werner & Smith’s work among high-risk youth [23,24]. Luthar, Cicchetti, & Becker’s critical appraisal of the concept of resilience led to its early definition as “a dynamic process encompassing positive adaptation within the context of significant adversity” [25]. A more recent conceptual analysis by Windle (2011) refined the notion of resilience as “the process of effectively negotiating, adapting to, or managing significant sources of stress or trauma. Assets and resources within the individual, their life, and environment facilitate this capacity for adaptation and ‘bouncing back’ in the face of adversity” [26]. This definition presumes that resilience will vary across the life course. Windle’s analysis postulates three key features of resilience: 1) an individual must encounter adversity, 2) an individual must be able to adapt to the adversity and 3) as a result of this process, a negative outcome is avoided [26]. The present study explored the first two of these three features of resilience among CDDs working in Côte d’Ivoire and Uganda’s national lymphatic filariasis and onchocerciasis programs.

## Methods

### Study design and setting

This mixed-methods study consisted of a cross-sectional survey of CDDs involved in MDAs in Cote d’Ivoire and Uganda in 2018. The study was carried out within the Abidjan and N’Zi Iffou regions of Côte d’Ivoire and the Omoro and Zombo districts of Uganda (Table 1).

**Table 1:** Description of study sites.

Country	Region or district	Settlement type	Population size	Endemicity
Côte d’Ivoire	Abidjan	Urban	4,395,243^a^	LF only
	N’Zi Iffou	Rural	247,578^a^	LF & Onchocerciasis
Uganda	Omoro	Rural	176,600^b^	LF & Onchocerciasis
	Zombo	Rural	219,800^b^	LF & Onchocerciasis

^a^ Institut National de la Statistique de Côte d’Ivoire. Recensement Général de la Population et de l’Habitat. Abidjan, Côte d’Ivoire; 2014.

^b^ Uganda Bureau of Statistics. National Population and Housing Census. Kampala, Uganda; 2014.

### Study population, participants, and sampling

Eligible CDDs were those who were directly involved in carrying out MDA activities during the 2018 MDA in their respective regions/districts and countries. Participants were recruited by the research teams in Côte d’Ivoire’s National Program for the Control of Neglected Tropical Diseases with Preventive Chemotherapy (PNLMTN-CP) and the Vector Control Division (VCD) of Uganda’s Ministry of Health. A total of 260 CDDs, 130 from each country and 65 from each region or district, were purposefully selected for inclusion in our study.

### Ethical considerations

The protocol for this study was reviewed and approved by the Bruyère Research Institute Research Ethics Board (#M16-18-012). In Côte d’Ivoire, the protocol was reviewed and approved by la Ministère de la Santé et de l’Hygiène Publique—Comité National d’Éthique des Sciences de la Vie et de la Santé (086-18/SHP/CNESVS-km). In Uganda, the protocol was reviewed by the Institutional Review Committee of the Vector Control Division (VCS-REC/075) and approval was granted by the Uganda National Council for Science and Technology (SS 4276). All participants in this study were adults aged 18 years or older. Each participant was read an informed consent form by data collectors, which was then read and signed by the participant prior to commencing the survey interview.

### Data collection and management

CDDs were interviewed using a micronarrative survey administered by trained enumerators. The micronarrative surveys captured data about CDD demographics, knowledge, attitudes, and practices, as well as a micronarrative prompt on CDDs personal experiences with the most recent MDA. Micronarrative surveys ground the information collected in the survey in the individuals’ actual and reported experiences within MDA programs. These real-life experiences become the foundation from which participants frame their opinions and attitudes [13]. The micronarrative prompt: “Think about the last time you carried out your activities for the NTD program. Would you tell me about one experience you remember most from the last NTD campaign?” was used to solicit brief stories about CDDs’ personal experiences with the most recent MDA and to capture additional data relevant to exploring CDDs’ resilience. Following the micronarrative prompt, a series of closed questions pertaining to the experience shared by the respondent were asked to collect additional information about the story’s subject, location, outcome, and related emotions. The micronarrative survey also contained questions to assess volunteers’ length and degree of involvement with the MDA programs, their motivations for volunteering, their perceptions of the support they received for their work, their perceptions of their community’s participation in the program, the issues they commonly encountered in the field and their understanding of program protocols. These surveys have been used previously by members of the study team to explore and identify issues that arise within MDA programs [13].

Quantitative data about CDD resilience was captured though administration of the Connor-Davidson Resilience Scale 25 (CD-RISC-25) following the micronarrative survey. The CD-RISC-25 is a 5-factor, internationally validated scale for assessing personal capacity to positively adapt to adversity [27,28]. Participants rate their agreement with 25 statements, ranging from “Not true at all” (0) to “True nearly all the time” (4). Scores of 0–4 for each of the 25 items are then summed to produce a total score out of 100 for each individual, with higher scores reflecting a greater personal capacity to positively adapt to adversity.

The micronarrative survey, including the CD-RISC-25, was translated into the local languages commonly spoken within each region or district using a process of translation, back-translation and verification that met the standards for the CD-RISC-25’s developers [29].

Survey data were collected and managed using the Research Electronic Data Capture (REDCap) tool hosted at Emory University. CDDs’ micronarratives were captured in the local language of each study site and transcribed verbatim using REDCap. Transcripts were then exported and translated into English by members of the research team who were native speakers of the local languages used. English versions of the micronarrative data were uploaded to NVivo Pro version 11.4.1 for management and analysis.

### Data analysis

#### Quantitative

Differences in the distribution of survey responses across key variables of interest were ascertained using chi square or Fisher exact tests, where appropriate. For our quantitative analysis of CDD resilience, the mean CD-RISC-25 score and standard deviation of our sample was first computed. To explore factors related to CDDs’ resilience, univariable and multivariable analyses were conducted, with CD-RISC-25 scores used as a continuous outcome measure and variables from the micronarrative surveys pertaining to CDD characteristics and experiences during MDA used as categorical explanatory variables. An aggregate multiple linear regression model of data collected from CDDs in both countries was constructed to explore and identify potential associations between CDD characteristics and their experiences during MDA and their scores on the CD-RISC-25.After identifying country to be an important covariate in the aggregate model, *post-hoc* individual country-specific analyses were conducted to further explore whether specific associations between CDDs’ characteristics or experiences and their scores on the CD-RISC-25 scale could be identified within each country.

#### Model building

Separate unadjusted linear regression analyses were used to determine the β-coefficients, 95% CI and *P*-values to test each explanatory variable’s association with the outcome. Explanatory variables with *P*-values <0.05 were then included in a full multiple linear regression model to determine which were independently associated with CDDs’ CD-RISC-25 scores. β-coefficients, 95% CI and *P*-values for each covariate in the multiple linear regression model were computed. A process of iteratively removing covariates from the model to increase the level of significance was undertaken until only significant covariates remained in the model. Exceptions to this process were made if removing a covariate reduced model fit, as determined by calculation of the Akaike information criterion (corrected; AICc). AICc is a measure of model fit, estimating maximum likelihood while adjusting for the number of parameters included to prevent model overfitting, even in small samples [30]. All quantitative analysis was conducted using STATA 14.0.

#### Qualitative

Thematic analysis was conducted using the micronarratives captured by the survey. This analysis was conducted following Ritchie et al.’s framework [31]. Themes related to the primary outcome of CDD resilience, including adversity encountered during work and support for CDDs, were identified. Coding was initially performed by one author independently then followed by discussion and development of themes and sub-themes together with authors in an iterative process. All qualitative analysis was conducted using NVivo version 11.4.1.

## Results

### Demographic characteristics of surveyed CDDs

Data from 248 completed CDD questionnaires, 132 from Côte d’Ivoire and 116 from Uganda, were included for quantitative analysis. Of 248 CDDs, 158 (63.7%) were male and 90 (36.3%) were female (Table 2). The majority of CDDs were 35 years or younger, and all had attained at least a primary school education. Differences in gender, age, education level, primary occupation and primary income source were observed across sites (Table 2).

**Table 2:** Demographics of surveyed community drug distributors (CDDs), by region/district.

Variable	Côte d’Ivoire				Uganda					
	Abidjan (N = 68)		N’Zi Iffou (N = 64)		Omoro (N = 63)		Zombo (N = 53)		Total (N = 248)	
	N	%	N	%	N	%	N	%	N	%
**Gender**										
Male	38	56.00%	46	71.90%	34	54.00%	40	75.50%	158	63.70%
Female	30	44.00%	18	28.10%	29	46.00%	13	24.50%	90	36.30%
**Age**										
18-25	23	33.80%	6	9.40%	4	6.40%	8	15.10%	41	16.50%
26-35	27	39.70%	23	35.90%	21	33.30%	23	43.40%	95	37.90%
36-45	16	23.50%	19	29.70%	21	33.30%	12	22.60%	69	27.40%
46-55	2	2.90%	14	21.90%	9	14.30%	9	17.00%	34	13.70%
56+	0	0.00%	2	3.10%	8	12.70%	1	1.90%	11	4.40%
**Education level**										
No education	0	0.00%	0	0.00%	0	0.00%	0	0.00%	0	0.00%
Primary school	0	0.00%	17	26.60%	27	42.80%	21	39.60%	65	26.20%
Secondary school: 1^st^ cycle	10	14.70%	22	34.40%	0	0.00%	0	0.00%	32	12.90%
Secondary school: 2^nd^ cycle	31	45.60%	16	25.00%	34	54.00%	31	58.50%	112	45.20%
Post-secondary education	27	39.70%	9	14.10%	2	3.20%	1	1.90%	39	15.70%
**Primary occupation**										
Agricultural activities	2	2.90%	27	42.20%	55	87.30%	51	96.20%	135	54.40%
Student	26	38.20%	4	6.30%	0	0.00%	1	1.90%	31	12.50%
Small scale enterprise	14	20.60%	12	18.80%	4	6.30%	0	0.00%	30	12.10%
Other	6	8.80%	9	14.10%	0	0.00%	0	0.00%	15	6.10%
No work	9	13.20%	2	3.10%	0	0.00%	0	0.00%	11	4.40%
Private employment	4	5.90%	4	6.30%	1	1.60%	0	0.00%	9	3.60%
Day worker	1	1.50%	1	1.60%	3	4.80%	1	1.90%	6	2.40%
Civil servant/government official	3	4.40%	3	4.70%	0	0.00%	0	0.00%	6	2.40%
Homemaker/housewife	3	4.40%	2	3.10%	0	0.00%	0	0.00%	5	2.00%
**Primary source of household income**										
Agricultural activities	6	8.80%	30	46.90%	53	84.10%	51	96.20%	141	56.60%
Small scale enterprise	21	30.90%	13	20.30%	7	11.10%	0	0.00%	41	16.50%
Civil servant/government official	23	33.80%	6	9.40%	0	0.00%	0	0.00%	29	11.70%
Other	7	10.30%	10	15.60%	0	0.00%	1	1.90%	18	7.30%
Private employment	7	10.30%	4	6.30%	1	1.60%	0	0.00%	12	4.80%
Day worker	4	5.90%	1	1.60%	2	3.20%	1	1.90%	9	3.20%

### Volunteerism among CDDs

The micronarrative questionnaire collected data on the length of time CDDs had been involved with the NTD program, as well as their involvement with other health programs. Most (73.4%) of the CDDs surveyed in our study had volunteered for one year or more, which varied by site (Table 3). Variations were also observed in the other disease control programs for which CDDs volunteered.

**Table 3:** Indicators of CDD volunteerism, by region/district.

Variable	Côte d’Ivoire				Uganda					
	Abidjan (N = 68)		N’Zi Iffou (N = 64)		Omoro (N = 63)		Zombo (N = 53)		Total (N = 248)	
	N	%	N	%	N	%	N	%	N	%
**Length of time volunteering in NTD program**										
<1 year	40	58.80%	13	20.30%	5	7.90%	8	15.10%	66	26.60%
1-3 years	22	32.40%	21	32.80%	21	33.30%	12	22.60%	76	30.70%
>3 years	6	8.80%	30	46.90%	37	58.70%	33	62.30%	106	42.70%
**Programs CDD has volunteered with over the past three years, apart from the NTD program**										
Malaria	52	76.50%	41	64.60%	34	54.00%	22	41.50%	149	60.10%
Polio/Immunization	61	89.70%	51	79.70%	7	11.10%	20	37.70%	139	56.10%
Maternal and child health	6	8.80%	13	20.30%	12	19.10%	15	28.30%	46	18.60%
None	7	10.30%	3	4.70%	22	34.90%	14	26.40%	46	18.60%
HIV/AIDS	5	7.40%	10	15.60%	8	12.70%	13	24.50%	36	14.50%
Other	0	0.00%	6	9.40%	9	14.30%	1	1.90%	16	6.50%

### CDD training and activities prior to, and during, MDA

Assessment of CDD training revealed that large proportions of CDDs were trained in various MDA activities, ranging from training on distributing the medications (94.8%) to training on what to do if someone refused to participate (70.2%) (Table 4). CDDs most frequently reported participating in distributing medication (96.0%) with fewer (19.8%) reporting that they tracked or following up on side effects during MDA. Notably, a third of CDDs said they completed reports during MDA, with significant variation between and within countries.

**Table 4:** CDD training and activities carried out during MDA, by region/district.

Variable	Côte d’Ivoire				Uganda					
	Abidjan (N = 68)		N’Zi Iffou (N = 64)		Omoro (N = 63)		Zombo (N = 53)		Total (N = 248)	
	N	%	N	%	N	%	N	%	N	%
**Activities CDD received training for**										
How to distribute medication/use measuring stick	67	98.50%	63	98.40%	54	85.70%	51	96.20%	235	94.80%
Techniques to encourage community participation	62	91.20%	59	92.20%	49	77.80%	40	75.50%	210	84.70%
Reports and registration	52	76.50%	44	68.80%	46	73.00%	51	96.20%	193	77.80%
What to do if there are side effects	53	77.90%	56	87.50%	35	55.60%	35	66.00%	179	72.20%
What to do when someone refuses to participate	54	79.40%	53	82.80%	39	61.90%	28	52.80%	174	70.20%
**Activities CDD participated in during MDA**										
Distributing medications	64	94.10%	62	96.60%	62	98.40%	50	94.30%	238	96.00%
Measuring community members	58	85.30%	59	92.20%	57	90.50%	46	86.80%	220	88.70%
Encouraging community to participate	56	82.40%	49	76.60%	62	98.40%	38	71.70%	205	82.70%
Registration	48	70.60%	36	56.30%	54	85.70%	52	98.10%	190	76.60%
Reporting	4	5.90%	12	18.80%	23	36.50%	33	62.30%	72	29.00%
Mopping up after MDA finished	4	5.90%	5	7.80%	24	38.10%	30	56.60%	63	25.40%
Tracking or following up on side effects	1	1.50%	12	18.80%	17	27.00%	19	35.90%	49	19.80%

### Reported challenges, stressors and adversity encountered by CDDs during MDA

In the micronarrative survey, CDDs were prompted to share a story about their most memorable experience from the previous MDA. Thematic analysis of these qualitative data led to five emergent themes: CDDs’ Evaluation of the Program, CDDs’ Experience with Disease and the Medication, Challenges Faced by CDDs, Community Response to Program, and Support for CDDs. A breakdown of these themes and their related sub-themes is provided in Table 5. Elements of CDD micronarratives were occasionally coded to multiple themes if they met the descriptions of two or more themes.

**Table 5:** Resultant themes and sub-themes from thematic analysis of micronarrative data.

Theme	Description
**1. CDDs’ Evaluation of Program**	Any stories that contained an evaluation of various components of the program.
	**Sub-themes:** Benefits of Volunteering, Evaluation of the Distribution, Self-Evaluation of Performance
**2. CDDs’ Experience with Disease and Medication**	Any experiences shared by CDDs about interacting with individuals with disease and any observations they made regarding the effects of the medication.
	**Sub-themes:** Experience with Disease, Experience with Medication
**3. Challenges Faced by CDDs**	Any mention of challenges faced by volunteers during MDA.
	**Sub-themes:** Challenges Related to Community Participation, Personal Challenges, Programmatic Challenges
**4. Community’s Response to Program**	General comments made about how the community has responded to the program. Coded references are about how the community members themselves perceive the program in their community.
	**Sub-themes:** Community Participation, Community’s Beliefs About Program, Community’s Trust in Program, Demands Made of Program, Emotional Response to Program
**5. Support for CDDs**	Any mention of support the volunteers received during MDA. Sub-themes are divided based on the source of this support
	**Sub-themes:** Support from Community Leaders, Support from Community Members, Support from Health Staff, Support from Supervisor

CDDs shared stories about some of the positive experiences they had during MDA, as well as some of the challenges they faced in their work. The number and types of challenges encountered by CDDs varied by country and by district/region. In the Abidjan region of Côte d’Ivoire, challenges appeared to stem from active community refusals due to political beliefs, misconceptions about medication safety and/or lack of information about the distribution:

*“People are chasing us because they say they have never heard of these drugs*, *or they say that the government gave the drugs to kill us*.*”*–CDD, Abidjan

*“The refusal of a doctor saying that the drug is to reduce the fertility of women*. *I myself have to drink to reassure them*.*”–*CDD, Abidjan

*“There was a case of a gentleman who called me all these names and he told me that he was not a guinea pig for the experiments of the white men to take these drugs*. *I was so shocked that I called my supervisor*. *I had been warned that the distribution of drugs was difficult*, *but I did not imagine that it was at this point*.*”–*CDD, Abidjan

The types of challenges encountered by CDDs in the N’Zi Iffou region of Côte d’Ivoire also included active community refusals, but these appeared to be due to fear of side effects and miscommunication or lack of awareness:

*“I was kicked out of a household because a year ago during one of the distributions the head of household had side effects that drove him to the hospital and caused expenses*.*”–*CDD, N’Zi Iffou

*“We were insulted by the population because for them it is a sterilizing drug and some people asked not to take it*.*”–*CDD, N’Zi Iffou

*“There is a young man from the village who insulted me when I presented him the drugs and refused to take them*.*”–*CDD, N’Zi Iffou

Also mentioned in a story from a CDD from the N’Zi Iffou region was a case where a pregnant women hid her pregnancy from the distributors:

*“A woman who was in early pregnancy has hidden her condition; she has drank the drugs and the pregnancy has passed*.*”–*CDD, N’zi Iffou

Micronarratives from CDDs in the Omoro district of Uganda had the highest number of coding references attributed to the “challenges faced by CDDs” theme. Challenges described by CDDs working in this district included active community refusals, absent community members, conflict with community members and leaders, and resource shortages:

*“There are some households (a few though) I went to and they refused to swallow the drugs saying the drugs are harmful and meant to kill them*.*”–*CDD, Omoro

*“I got a drunkard during the time I was distributing the drugs and he became so aggressive*.*”–*CDD, Omoro

*“I was moving from house to house and this made me very tired*. *However*, *the exercise went on very well since most people took the drugs*. *However*, *some politicians were discouraging people from taking the drugs saying it would make people infertile or kill them*.*”–*CDD, Omoro

*“Sometimes I would go to distribute drugs and not find some people home*, *yet it is very tiresome to come back because the area is large*.*”*–CDD, Omoro

*“I distributed the drugs to most people*, *however some people did not receive the drugs since the drugs were too few and people thought we were just hiding the drugs”–*CDD, Omoro

Fewer challenges were mentioned by CDDs living in Zombo district (Uganda) and mostly pertained to the absence of community members during the distribution andthe physical demands of walking long distances within their large coverage areas.

*“During the last NTD program I moved to every household and all people managed to receive their pills*, *though there where some challenges I encountered like absence of the household members and long distance*.*”*–CDD, Zombo

*“It is tedious work since it involves moving house to house and mobilising the community*.*”–*CDD, Zombo

CDDs were asked how they remember feeling at the time of their story, serving to better contextualize the experiences they shared (Table 6). There was variation in perceptions of safety and happiness across the four districts and within the countries, with Omoro reporting the least feelings of safety across all sites.

**Table 6:** CDDs’ feelings at the time of their micronarrative story, by region/district.

Variable	Côte d’Ivoire						Uganda			
	Abidjan (N = 68)		N’Zi Iffou (N = 64)		Omoro (N = 63)		Zombo (N = 53)		Total (N = 248)	
	N	%	N	%	N	%	N	%	N	%
**CDD’s feelings at time of micronarrative**										
Happy	21	30.90%	26	40.60%	16	25.40%	40	75.50%	103	41.50%
Sad	34	50.00%	17	26.60%	18	28.60%	3	5.70%	72	29.00%
Did not share a story	0	0.00%	0	0.00%	15	23.80%	5	9.40%	20	8.10%
No emotion	8	11.80%	8	12.50%	3	4.80%	1	1.90%	20	8.10%
Other (frustrated, afraid, shocked, etc.)	3	4.40%	11	17.20%	2	3.20%	2	3.80%	18	7.30%
Angry	2	2.90%	2	3.10%	9	14.30%	1	1.90%	14	5.70%
Missing	0	0.00%	0	0.00%	0	0.00%	1	1.90%	1	0.40%
**CDD’s feelings of safety at time of micronarrative**										
Safe	54	79.40%	54	84.40%	34	54.00%	46	86.80%	188	75.80%
Somewhat safe	7	10.30%	4	6.30%	5	7.90%	0	0.00%	16	6.50%
Not safe	7	10.30%	6	9.40%	8	12.70%	1	1.90%	22	8.90%
Not applicable	0	0.00%	0	0.00%	1	1.60%	0	0.00%	1	0.40%
Did not share a story	0	0.00%	0	0.00%	15	23.80%	5	9.40%	20	8.10%
Missing	0	0.00%	0	0.00%	0	0.00%	1	1.90%	1	0.40%

Finally, to understand issues that CDDs encounter during MDA, the micronarrative questionnaire asked about the problems most commonly encountered (Table 7). These included difficulty reaching the community (45.6%), not having enough time to carry out personal tasks (30.2%) and not having enough time to carry out tasks for the NTD program (26.6%). The types of problems encountered by CDDs also varied across study sites.

**Table 7:** Distribution method and reported issues encountered by CDDs, by region/district.

Variable	Côte d’Ivoire				Uganda					
	Abidjan (N = 68)		N’Zi Iffou (N = 64)		Omoro (N = 63)		Zombo (N = 53)		Total (N = 248)	
	N	%	N	%	N	%	N	%	N	%
**Common problems encountered by CDDs during MDA**										
Difficult to reach the community	18	26.50%	13	20.30%	47	74.60%	35	66.00%	113	45.60%
Not enough time to carry out personal tasks	7	10.30%	20	31.30%	35	55.60%	13	24.50%	75	30.20%
Not enough time to carry out NTD program tasks	18	26.50%	13	20.30%	27	42.90%	8	15.10%	66	26.60%
Run out of supplies (drugs, educational materials)	12	17.70%	6	9.40%	22	34.90%	18	34.00%	58	23.40%
Too much responsibility/many tasks to do	11	16.20%	11	17.20%	13	20.60%	7	13.20%	42	16.90%
Community is not responsive to the NTD program	22	32.40%	13	20.30%	3	4.80%	0	0.00%	38	15.30%
Not enough results from the NTD program in my district	9	13.20%	13	20.30%	9	14.30%	4	7.60%	35	14.10%
Not enough supervision	1	1.50%	2	3.10%	22	34.90%	5	9.40%	30	12.10%
Planning is not well done	5	7.40%	7	10.90%	1	1.60%	5	9.40%	18	7.30%

### Support CDDs reported receiving during MDA

CDDs were asked the type of support (tangible or in-kind) received from the NTD program, other volunteers and from their communities during MDA and whether CDDs were supervised during the MDA and who supervised them (Table 8).

**Table 8:** Support CDDs reported receiving from their community and the NTD program, by region/district.

Variable	Côte d’Ivoire						Uganda			
	Abidjan (N = 68)		N’Zi Iffou (N = 64)		Omoro (N = 63)		Zombo (N = 53)		Total (N = 248)	
	N	%	N	%	N	%	N	%	N	%
**CDD received something from the NTD program for the activities carried out during the last MDA**										
Yes	65	95.6%	61	95.3%	18	28.6%	52	98.1%	196	79.0%
No	3	4.4%	3	4.7%	45	71.4%	1	1.9%	52	21.0%
**Community offered CDD support for their work during the last MDA**										
Yes	36	52.9%	38	59.4%	14	22.2%	20	37.7%	108	43.6%
No	32	47.1%	26	40.6%	49	77.8%	33	62.3%	140	56.5%
**Individuals CDDs reported supporting them in their work**										
CDD was supported by other NTD volunteers in their community	66	97.1%	58	90.6%	42	66.7%	49	92.5%	215	86.7%
CDD was supported by health workers in their community	27	39.7%	49	76.6%	11	17.5%	24	45.3%	111	44.8%
CDD was supported by leaders in their community	10	14.7%	35	54.7%	19	30.2%	46	86.8%	110	44.4%
No one; CDD worked alone most of the time	0	0.0%	10	15.6%	25	39.7%	1	1.9%	36	14.5%
**Who supervised CDD during their participation in the MDA**										
Parish supervisor	0	0.0%	0	0.0%	29	46.0%	48	90.6%	78	31.5%
Nurse/midwife	17	25.0%	55	85.9%	1	1.6%	0	0.0%	73	29.4%
District health authority staff/inspector	33	48.5%	16	25.0%	0	0.0%	15	28.3%	64	25.8%
Community supervisor	26	38.2%	6	9.4%	8	12.7%	20	37.7%	60	24.2%
CDD was unsupervised	0	0.0%	0	0.0%	25	39.7%	3	5.7%	28	11.3%
Village leader	0	0.0%	0	0.0%	3	4.8%	22	41.5%	25	10.1%
Other	3	4.4%	0	0.0%	5	7.9%	1	1.9%	9	3.6%
Doctor	0	0.0%	3	4.7%	0	0.0%	0	0.0%	3	1.2%

In general, CDDs reported receiving some form of support or compensation for their work from the NTD program within which they work. The exception was CDDs in Omoro district in Uganda, where 71.4% of CDDs reported not receiving anything. In contrast to the support CDDs received from the NTD program, only 43.6% of CDDs reported being offered support from the community for their work, ranging from 59.4% of CDDs from N’Zi Iffou to only 22.2% of CDDs from Omoro. Variation was also seen between and within countries with respect to the individuals who supported and supervised CDDs during MDA.

### CDDs’ personal capacity to overcome adversity

The CD-RISC-25 scale was analyzed to explore CDDs’ personal capacity for overcoming adversity. A summary of the aggregate mean ± SD CD-RISC-25 score for the study sample, as well as the mean CD-RISC-25 scores for each district, is presented in Table 9.

**Table 9:** Summary of CDDs’ mean CD-RISC-25 scores ± SD, by region/district.

Variable	Côte d’Ivoire		Uganda		
	Abidjan (N = 68)	N’Zi Iffou (N = 64)	Omoro (N = 63)	Zombo (N = 53)	Total (N = 248)
	Mean ± SD	Mean ± SD	Mean ± SD	Mean ± SD	Mean ± SD
**CD-RISC-25 Score**	73.3 ± 15.5	71.3 ± 14.5	77.6 ± 9.5	82.7 ± 10.8	75.9 ± 13.5

The overall mean ± SD CD-RISC-25 score for our sample of CDDs was determined 75.9 ± 13.5. Chronbach’s α, calculated to test the scale’s internal consistency, was 0.89, indicating that the scale demonstrated excellent internal reliability in our study. The results of our multiple linear regression analysis of potential associations between CDDs’ responses to the categorical explanatory variables on the micronarrative survey and CDDs’ scores on the CD-RISC-25 are presented in Table 10.

**Table 10:** Correlates of CDDs’ scores on the CD-RISC-25 scale.

Variable	N	Adjusted Model Mean Coeff. (95% CI) p-value			
**Country**					
Côte d’Ivoire	132	72.4	REF		
Uganda	116	80.0	5.74	(2.15 ─ 9.34)	0.002
**Volunteer received feedback on their work**					
No	85	73.6	REF		
Yes	163	77.1	2.70	(-0.68 ─ 6.08)	0.117
**CDD was supervised by a village leader**					
No	223	74.1	REF		
Yes	25	92.5	12.42	(6.92 ─ 17.92)	<0.001
**CDD reported being offered some form of support from the community**					
No	140	73.6	REF		
Yes	108	79.0	5.76	(2.66 ─ 8.86)	<0.001
**CDDs’ self-reported relationship with community**					
Not an easy relationship	35	66.9	REF		
Easy relationship	213	77.4	8.48	(4.20 ─ 12.76)	<0.001
**CDD defines their success as a volunteer as being able to use the skills they were taught**					
No	121	78.1	REF		
Yes	127	73.8	-5.07	(-7.96 ─ -2.17)	0.001

Our aggregate analysis revealed that CDDs from Côte d’Ivoire generally scored lower on the CD-RISC-25 than their counterparts in Uganda (*P* = 0.002). CDDs who said they were supervised by a village leader during MDA scored higher on the CD-RISC-25 than those who did not (*P*<0.001). CDDs who said that their communities offered them some form of support during the MDA tended to have higher CD-RISC-25 scores than those who did not (*P*<0.001). Similarly, those CDDs who reported having an “easy” relationship with their communities scored higher on the scale than those who reported having an “average” or “difficult” relationship with their communities (*P*<0.001). Finally, CDDs who qualified their success as a volunteer as being able to use the skills that they were taught tended to score lower on the CD-RISC-25 than volunteers who made no such qualification (*P* = 0.001). Whether or not CDDs received feedback on their performance during MDA improved the fit of the aggregate model but was not significantly associated with CD-RISC-25 scores. Age, sex, education level, primary occupation, and length of time spent volunteering with the NTD program were not significantly associated with CDDs’ CD-RISC-25 scores.

Given the significance of the country covariate in the aggregate model and previous observations of regional/contextual differences in CD-RISC-25 scores, we constructed country-specific multiple linear regression models to further explore potential associations between the characteristics of CDDs, their experiences during MDA and their CD-RISC-25 scores. (Tables 11 and 12)

**Table 11:** Correlates of CD-RISC-25 scores for CDDs volunteering in MDA programs in Côte d’Ivoire.

Variable	N	Adjusted Model Mean Coeff. (95% CI) p-value			
**CDD was supervised by a community supervisor**					
No	100	75.2	REF		
Yes	32	63.0	-9.63	(-14.83 ─ -4.44)	<0.001
**Common problem - not enough time to carry out tasks for the NTD program**					
No	101	70.3	REF		
Yes	31	79.1	6.20	(0.88 ─ 11.52)	0.023
**CDD reported receiving some form of support from the community**					
No	58	68.1	REF		
Yes	74	75.7	5.41	(0.82 ─ 10.01)	0.021
**CDD also volunteers for maternal and child health programs**					
No	113	73.4	REF		
Yes	19	65.8	-7.68	(-13.91 ─ -1.45)	0.016
**Relationship between volunteer and community**					
Not an easy relationship	31	66.9	REF		
Easy relationship	101	74.0	6.59	(2.03 ─ 12.45)	0.007
**CDD defines success in their role as being able to use the skills they were taught**					
No	66	76.4	REF		
Yes	66	68.2	-6.57	(-11.18 ─ -1.97)	0.005

**Table 12:** Correlates of CD-RISC-25 scores for CDDs volunteering in MDA programs in Uganda.

Variable	N	Adjusted Model Mean Coeff. (95% CI) p-value			
**District**					
Omoro	63	77.6	REF		
Zombo	53	82.7	-2.82	(-5.38 ─ 0.92)	0.164
**Volunteer was chosen by community members**					
No	20	73.3	REF		
Yes	96	81.4	4.47	(0.79 ─ 8.14)	0.018
**CDD self-volunteered**					
No	94	77.9	REF		
Yes	22	88.6	4.51	(0.49 ─ 8.25)	0.028
**CDD was supervised by a village leader**					
No	91	76.5	REF		
Yes	25	92.5	11.47	(7.03 ─ 15.92)	<0.001
**Common problem - not enough time to carry out tasks for the NTD program**					
No	81	81.7	REF		
Yes	35	76	-4.96	(-8.11 ─ -1.81)	0.002
**CDD reported receiving some form of support from the community**
No	82	77.4	REF		
Yes	34	86.1	3.13	(-0.16 ─ 6.42)	0.062
**CDD does not volunteer for any other health program**					
No	80	81.7	REF		
Yes	36	76.2	-3.73	(-6.76 ─ -0.70)	0.016

The multiple linear regression analysis of CD-RISC-25 scores among CDDs in Côte d’Ivoire showed that CDDs reporting they were supervised by a community supervisor during MDA scored lower on the CD-RISC-25 than CDDs who did not (*P*<0.001). CDDs who said that one of the common problems they faced during MDA was that they did not have enough time to carry out their tasks for the MDA program tended to have higher CD-RISC-25 scores than those who did not (*P* = 0.023). CDDs who said that they also volunteered for maternal and child health programs in their communities tended to score lower on the CD-RISC-25 than those who did not volunteer (*P* = 0.016). As in the aggregate analysis of CDDs’ CD-RISC-25 scores, CDDs who reported being offered some form of support by the community during MDA scored higher on the CD-RISC-25 score than those who did not (*P* = 0.021). Similarly, CDDs who reported having “easy” relationships with their communities tended to score higher on the CD-RISC-25 than those who reported having “average” or “difficult” relationships. Finally, as in the aggregate analysis, CDDs who said that they knew they were successful in their role as a volunteer because they were able to use the skills they were taught also were volunteers who scored lower on the CD-RISC-25. As with the aggregate analysis, age, sex, education level, primary occupation, and length of time spent volunteering with the NTD program were not significantly associated with CDDs’ CD-RISC-25 scores.

The Uganda-specific multiple linear regression analysis found different factors to be associated with CDDs’ scores on the CD-RISC-25 when compared to the analysis of CD-RISC-25 scores from CDDs in Côte d’Ivoire. The analysis found no difference between the mean CD-RISC-25 scores of CDDs in Omoro and Zombo districts (*P* = 0.164). The ‘district’ variable was left in the model as its inclusion improved model fit. CDDs who reported being offered some form of support from their community during MDA appeared to score higher on the CD-RISC-25. As with the ‘district’ variable, however, this association was not statistically significant (*P* = 0.062) and left in the model as its removal reduced model fit. All remaining covariates in the model were significantly associated with CDDs’ CD-RISC-25 scores. CDDs who were either selected by their communities to volunteer or who self-volunteered for the NTD program scored higher than CDDs who were not (*P* = 0.018 and *P* = 0.028, respectively). CDDs who reported that they were supervised by a village leader during MDA scored higher on the CD-RISC-25 than those who did not (*P* < 0.001). Covariates associated with CDDs scoring lower on the CD-RISC-25 were if the CDD reported not having enough time to carry out their tasks for the NTD program (*P* = 0.002) and if the CDD did not volunteer for any other health program in their community *(P* = 0.016). As observed in the aggregate and Côte d’Ivoire-specific analysis of CDDs’ CD-RISC-25 scores, age, sex, education level, primary occupation, and length of time the CDD had volunteered with Uganda’s NTD program were not significantly associated with their scores on the scale.

## Discussion

CDDs have been considered to be crucial to global efforts to control or eliminate NTDs [8,17,32] yet a better understanding and exploration of CDDs’ experiences within NTD programs has been called for, especially amid intensified elimination agendas at the local, national and international levels [33]. To our knowledge, ours is the first study that has explored the concept of psychological resilience among CDDs working within NTD programs.

A high-level summary of the factors associated with resilience of CDDs working in Côte d’Ivoire and Uganda is provided in Box 1. Our discussion of the results is divided into three parts: identity of CDDs and how this intersects with their experiences in the MDA program; contextualization of the adversity reported by CDDs during MDA; interpretation of CDDs’ resilience using the CD-RISC-25.

**Box 1. Summary of key findings**

- The profiles of CDDs can vary considerably between rural and urban settings
- CDDs encounter diverse programmatic, interpersonal, and personal challenges in their work, both between and within national- and community-level contexts.
- Community support, ownership, and supervision of NTD activities, as well as community-volunteer relationships, may be an important factor for fostering CDD resilience
- CDDs in rural settings commonly volunteer for multiple health programs, highlighting the potential for resilience-promoting interventions to have cross-cutting impacts
- The CD-RISC-25 is a reliable and useful tool for future research concerning CDD resilience

### Characteristics of CDDs

The analysis revealed that the characteristics of urban and rural CDDs were different, particularly in Côte d’Ivoire. CDDs in rural communities typically are older, male farmers with lower levels of formal education and have volunteered for the NTD program longer than urban CDDs. Because of these differences, those in urban environments are expected to have different experiences within the NTD program, encounter different challenges, and have different supports available to overcome these challenges than their counterparts in rural areas. Other studies have found urban-specific challenges encountered by CDDs [34,35]. In keeping with the global strategies to accelerate programmatic action, interventions to better support CDDs should take into consideration the different challenges faced by CDDs in urban and rural environments.

CDDs in rural communities across Côte d’Ivoire and Uganda can be considered almost as “professional” volunteers. CDDs in these communities often volunteer with NTD programs for many years, in addition to other health programs that take place in their communities. This finding can be leveraged as an opportunity for health system strengthening; e.g., efforts taken by NTD programs to bolster CDDs’ resilience can have positive impacts on program success that extend horizontally across other health programs, as well as forward in time into future iterations of the NTD program. Integration of a well-supported and well-trained CDD workforce as a way to improve health system capacity and support other health programs has been suggested by other studies as well [36–38]. Investment in this area would contribute to the WHO 2030 NTD roadmap goal on intensifying cross-cutting approaches.

### Adversity encountered by CDDs

CDDs face a range of challenges, including conflict, threats, and other stressors. Interpersonal conflict occasionally arose between CDDs and community members. The reasons for this varied, but most appeared to be related to community members not trusting the CDDs who were distributing the drugs, the drugs themselves, or the program as a whole. CDDs in our study shared stories about being chased from homes or insulted and even threatened, situations which are not unique to this research [33,39]. In Côte d’Ivoire we noted that politics played a role in community members’ distrust of the CDDs and the MDA. Interpersonal conflict was also encountered by CDDs in Uganda, particularly those working in Omoro district. Some CDDs here reported conflict with individuals under the influence of alcohol while others reported conflicts with community members who did not know who they were or who did not trust them. Many CDDs in Omoro reported difficulties finding community members during the distribution. This disproportionately high number of challenges in Omoro compared to the other sites may be explained by recent history of armed conflict in the district [40]. CDDs also can encounter moral dilemmas while interacting with community members; a story about the accidental treatment of a pregnant woman who did not disclose her pregnancy during MDA being a prime example.

CDDs commented on the massive workload associated with distributing the medicines house-to-house and how physically exhausting this work could be. This finding is unsurprising, as CDDs in rural communities are often assigned large coverage areas and are required to walk kilometers to reach their assigned households, a finding documented elsewhere [41,42]. Some of the CDDs commented on the opportunity costs associated with participating in the program. In particular, those in rural regions/districts commented that because of their commitments to the NTD program, they were unable to work on their farms or participate in other income-generating activities. Other studies have noted that due to low remuneration in the NTD program, CDDs incur substantial opportunity costs when they volunteer [43–45]. Many CDDs reported having not enough time to carry out both their tasks for the NTD program or their own personal tasks. The combination of a heavy workload and the opportunity costs associated with being a CDD risks compounding the stress they may experience balancing their programmatic and personal commitments.

CDDs encountering adversity in their work can have implications for the programs and communities they serve. CDD performance is an important predictor of MDA success [14,15]. CDD performance is hindered when they encounter difficult situations for which they have not been trained or are left unsupported to address. When CDDs encounter stressors or adversity that they feel incapable of navigating, it can impact their performance over the course of MDA and can even lead to their eventual withdrawal from the program. CDD attrition can undermine the capacity and effectiveness of NTD programs. Thus, from a programmatic standpoint, attention needs to be given to addressing the realities that CDDs face and their subsequent needs [17,46]. More importantly, the adversity encountered by CDDs has implications for the well-being of CDDs themselves. As CDDs are often members of the communities they serve, the impact of conflicts and stressors they encounter during MDA risk to persist after the program’s conclusion. National and global NTD initiatives rely on the altruism of CDDs to carry out programming. As such, care should be taken to ensure that CDD involvement in such programs is done in an ethical manner; one that properly informs CDDs of the demands of the work, prepares them for it, and has strategies in place to mitigate risks to their physical or mental well-being.

### CDD resilience in the face of adversity

Given the evidence that CDDs encounter multiple types stressors in their work, we used the CD-RISC-25 to understand their personal capacity to cope with these stressors (i.e., resilience). Understanding CDDs’ resilience in the face of adversity is an important part of addressing any negative effects that may stem from it. CDDs’ resilience is contingent upon their social and physical environment, the tangible and intangible resources available to them, and their own personal characteristics or capacities that can aid or hinder them throughout this process [26].

As observed in our aggregate analysis of CD-RISC-25 scores, CDDs who had good relationships with their community, received support from the community for their work, or were supervised by community leadership during MDA scored higher on the CD-RISC-25 than those who did not. This suggests that involving members of the community and community leadership in the development and implementation of NTD programs through a community-directed approach may also have implications for the psychological well-being of the volunteers who deliver such programs. In the available literature on resilience, social support and interpersonal relationships have been shown to be a crucial factor in one’s ability to adapt to stress or adversity [47–49]. Notably, these are all factors that may also influence CDDs’ performance and motivation and are also all integral components of the community-directed treatment (CDT) approach [16,50–52]. This evidence suggests a renewed emphasis on the CDT approach to MDA could be beneficial for CDD performance, motivation and psychological well-being. Interestingly, our aggregate analysis found no associations between CDDs’ scores on the CD-RISC-25 and their gender, age, education level, primary occupation, or the length of time they have spent volunteering with the NTD program. While the lack of association between demographic characteristics and resilience has been found in other studies, it should be noted that demographic characteristics can affect individuals’ access to resources and support and should be investigated further in future studies of CDD resilience [53].

The results of our analysis revealed that CDDs’ scores on the CD-RISC-25 differed significantly between countries, which was not surprising given that the characteristics, environments, and experiences of CDDs differ substantially. This finding supports the notion that resilience is highly contextual and may be difficult to compare across countries, cultures and programs [49]. As such, between-country comparisons may not be as informative as within-country comparisons and, any intervention to improve CDD resilience should be tailored accordingly. Despite this, it appears as though certain factors may be broadly related to CDD resilience across contexts, including community support for CDDs’ work, CDDs’ relationships with their communities, involvement of community leadership, and their personal definitions of success.

### Country-specific considerations for CDD resilience

In our country-specific analyses, we observed how different factors may be related to CDDs’ resilience. In Côte d’Ivoire there was notably no difference between the CD-RISC-25 scores of volunteers from the urban region of Abidjan and the rural region of N’Zi Iffou. Our analysis also indicated that factors related to program structure, delivery, and social environment were associated with CDDs’ scores. CDDs who said that they were supervised by a community supervisor tended to score lower on the CD-RISC-25, suggesting that the type of supervision has implications for CDD well-being. CDDs’ other volunteer experience may also influence well-being during MDA, as we found that CDDs from Côte d’Ivoire who also volunteered for maternal and child health programs appeared to score lower on the CD-RISC-25. Volunteering for many demanding health programs has the potential to lead to CDDs over-extending themselves. It is also possible that CDDs carry conflict or challenges encountered in other programs into the NTD program. Interestingly, CDDs in Côte d’Ivoire who said that there was not enough time to carry out their tasks for the program scored higher on the CD-RISC-25 than those who did not, the inverse of what was found in Uganda. The reasons for these differences are unclear and require further exploration.

In Uganda, our analysis noted that volunteers chosen by members of the community scored higher on the CD-RISC-25. As previously mentioned, involving communities in the selection of CDDs could help establish bonds of trust, help CDDs feel supported in their participation in MDA, and mitigate potential conflicts, as community members will be familiar with the people who provide them with the medication. Aside from potentially bolstering CDDs’ resilience, community selection of CDDs has been previously emphasized as being vital for sustaining community support for MDA and shaping community participation in the intervention [54]. Despite the repeatedly demonstrated benefits and successes of community-directed treatment distribution, communities are not always engaged in the planning and shaping of MDA implementation or the selection of CDDs [41,42,52]. Efforts to re-engage communities in the selection process could have implications for the well-being of CDDs in Uganda.

CDDs in Uganda who self-volunteered for the program tended to have higher CD-RISC-25 scores. It is possible that these individuals possessed personal characteristics, such as confidence and a strong sense of self-efficacy, which led them to feel more comfortable volunteering for the program and prepared to handle the challenges associated with it. CDDs who reported that they did not volunteer for any other health program in their communities tended to score lower on the CD-RISC-25. It is possible that prior or concurrent experience as a community health volunteer equips individuals with the capacity to navigate stressors encountered in the NTD program better than those who have no such experience. The CDD selection process should ensure that selected individuals are adequately prepared and supported to deal with the challenges inherent to the role. CDDs in Uganda who were supervised by village leaders had higher CD-RISC-25 scores than those who did not, suggesting that the involvement of community leadership in MDA may similarly have positive implications for the well-being of the CDDs working in their communities. Involving community leaders in MDA design and implementation may have beneficial impacts on MDA coverage and compliance [32,54]. Our results suggest that involvement of community leadership in CDD selection and supervision may also positively affect CDD well-being.

A final takeaway from our exploration of CDD resilience in Uganda was the observation of differences in the experiences of CDDs from each district, despite volunteering within the same NTD program. Volunteers in Omoro generally encountered more challenges and reported receiving less support than their counterparts in Zombo, and they had lower CD-RISC-25 scores, although this difference was not statistically significant in the multiple linear regression model. Omoro is recovering from recent armed conflict and is inhabited by a high proportion of internally displaced people; as such special care should be taken to understand the experiences and challenges encountered by CDDs in areas of conflict or civil unrest, and to support their resilience, as NTDs are likely to persist in these areas beyond target dates for current elimination efforts [40,55].

To our knowledge, this was the first study that assessed resilience in CDDs using the CD-RISC-25. This widely used measure exhibited a high degree of internal reliability in our study. CDDs’ CD-RISC-25 scores do not appear to differ substantially from those in other study populations. For example, a national random digit dial sample of residents in the United States reported a mean CD-RISC-25 score of 80.4 ± 12.8 [27]. Studies looking at the CD-RISC-25 scores of clinical associates in South Africa, medical interns in the United States, and nurses in the United States revealed mean scores of 77.4, 75.3 and 66.5, respectively [56–58]. While resilience is thought to be highly contextual [49], the similarity between our results and the results of other studies places the mean CD-RISC-25 scores of CDDs in proximity to the mean scores of other health worker populations, where stressors may be similar. However, for more meaningful comparisons to be drawn, further studies that incorporate the use of the CD-RISC-25 to survey CDDs and other community health volunteers are warranted. Future research should additionally focus on assessing Windle’s third component of resilience among CDDs, to determine whether resilience aids CDDs in avoiding negative outcomes that may result from program-related stressors, including volunteer attrition, personal expenditures, or diminished physical, psychological, and emotional well-being [26].

## Conclusion

This study lays the groundwork for future research into the psychological well-being of volunteer CDDs carrying out MDAs for the elimination of PC-NTDs. The study demonstrated that lack of involvement of leadership or support from communities during MDA could pose possible risks to CDDs’ resilience. Positive community-CDD relationships may help CDDs adapt to the challenges they encounter in their work. Follow-up studies should examine changes in CDD resilience over time, particularly prior to, during and post-MDA, and which factors are predictive of positive adaptation to the adversity they may encounter in their work. The CD-RISC-25 demonstrated good reliability in our study and can be a useful tool for measuring CDDs’ personal capacity to overcome adversity in such research. Other factors that should be explored further include CDDs’ relationships with their communities, whether community members and community leaders supported them in their work, who supervised them, how they were selected, and their aspirations and motivations as a volunteer. Potential outcome measurements could include whether CDDs returned to volunteer in subsequent MDAs or culturally appropriate measures of psychological well-being. Future research should further explore the professional experiences and resilience of CDDs, not only to sustain the gains made by global NTD elimination efforts and progress towards the WHO 2030 NTD road map, but to ensure that CDD involvement in these efforts is done with their safety and well-being in mind.

## Supporting information

S1 Questionnaire PLOS questionnaire. (PDF)
